# Designing Reliable Cathode System for High‐Performance Inorganic Solid‐State Pouch Cells

**DOI:** 10.1002/advs.202401889

**Published:** 2024-03-30

**Authors:** Shuying Wang, Sheng Liu, Wei Chen, Yin Hu, Dongjiang Chen, Miao He, Mingjie Zhou, Tianyu Lei, Yagang Zhang, Jie Xiong

**Affiliations:** ^1^ School of Materials and Energy University of Electronic Science and Technology of China Chengdu 610054 China; ^2^ State Key Laboratory of Electronic Thin Films and Integrated Devices University of Electronic Science and Technology of China Chengdu 610054 China

**Keywords:** all‐solid‐state battery, cathode materials, inorganic solid‐state electrolyte, solid‐state pouch cell

## Abstract

All‐solid‐state batteries (ASSBs) based on inorganic solid electrolytes fascinate a large body of researchers in terms of overcoming the inferior energy density and safety issues of existing lithium‐ion batteries. To date, the cathode designs in the ASSBs achieve remarkable achievements, adding the urgency of scaling up the battery system toward inorganic solid‐state pouch cell configuration for the application market. Herein, the recent developments of cathode materials and the design considerations for their application in the pouch cell format are reviewed to straighten out the roadmap of ASSBs. Specifically, the intercalation compounds and the conversion materials with conversion chemistries are highlighted and discussed as two potentially valuable material types. This review focuses on the basic electrochemical mechanisms, mechanical contact issues, and sheet‐type structure in inorganic solid‐state pouch cells with corresponding perspectives, thus guiding the future research direction. Finally, the benchmarks for manufacturing inorganic solid‐state pouch cells to meet practical high energy density targets are provided in this review for the development of commercially viable products.

## Graphical abstract

Outstanding cathode performance is a decisive condition for the transformation of inorganic solid‐state battery research from the laboratory level mold cell to the commercial applicable pouch cell. This review systematically retrospected the material and engineering design on cathode, aiming to provide benchmark and guidance for the future development of inorganic solid‐state pouch cell.

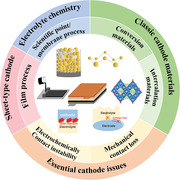

## 1 Introduction

The electric vehicle industry has been growing rapidly in recent years, allowing the continuous demand of lithium‐ion batteries (LIBs) to ensure adequate energy supply for electrical appliances.^[^ ^1^ ^]^ To date, the efforts to increase the safety of the LIBs with liquid electrolytes (LEs) have encountered bottleneck.^[^ ^2^ ^]^ Researchers have tried to improve the safety of batteries from many aspects such as electrolyte additives,^[^ ^3^ ^]^ separator modification,^[^ ^4^ ^]^ battery management systems, and so on.^[^ ^5^ ^]^ However, they still cannot guarantee the safety of liquid LIBs systems, especially for electric vehicles. Solid‐polymer electrolyte consisting of a homogeneous mixture of polymer and lithium salts, are flexible and soft with good ionic conductivity, which shows great potential in the commercial application. Still, more efforts should be given to eliminate the safety concern completely. In this context, substituting liquid and solid‐polymer electrolytes by all solid‐state inorganic electrolyte featuring with high temperature resistance and incombustibility, is expected to improve the safety of LIBs.^[^ ^6^ ^]^ On the other side, inorganic electrolyte membrane can be made thin enough with small risk of short circuit to improve energy density.

The basic architecture of all‐solid‐state batteries (ASSBs) is similar to that of liquid LIBs except that the liquid components are replaced by solid‐state electrolyte (SE) materials. Obviously, challenges such as poor solid contact, fragility, processing difficulty, and low ion transport rate are emerging, limiting capacity, rate, and cycling stability of ASSBs.^[^ ^7^ ^]^ Moreover, ASSBs assembled with mold cell format are currently mainstream solution in which the assemble pressures could be easily provided to 300 MPa to compact the electrode and electrolyte materials.^[^ ^8^ ^]^ Accordingly, considerable efforts for improving the battery performances of ASSBs such as long‐cycle, high‐rate, and high capacity had been made based on mold cell format.^[^ ^9^ ^]^ However, mold cell formed of steel column, PEEK sealing hood, ceramic sleeve, and stainless‐steel shell, has extremely low energy density, and is too complex to put into practical application. We need to notice that the prismatic cell may be a more suitable battery structure than pouch cell in terms of cell test because the tough aluminum shell has a unique advantage in maintaining external pressure during the region of 2–4 Mpa. However, it still has a big challenge for the application in all inorganic‐solid‐state batteries according to the current research progress as considering the following factors: First, the sheets stacking method is the only way based on current technology to assemble the all‐solid‐state batteries. The production of all inorganic solid electrolyte film is mainly based on dry PTFE fibrosis or wet casting, resulting in the poor flexibility for the rolled and flattened assembly method. Then, the requirement for ultra‐high pressure (>300 MPa) hot (or warm) isostatic pressure for manufacturing ceramic‐based all‐solid‐state batteries leads to the inevitable use of plastic film to encapsulate the cell core as a pouch cell, because the cell should be completely immersed by water or oil medium to ensure uniform pressure. Therefore, packaging the pouch cells in the prismatic structure may be a better solution for the latter to ensure the applied pressure of 2–4 MPa during test. Overall, pouch cell seems to be a reasonable way to make a cell, and prismatic cell is rather a reasonable structure for testing. Pouch cell is the final cell format for the actual application of ASSBs, gradually attracting the board of academic community.^[^ ^10^ ^]^ However, the design of pouch cells is particularly complex. Only few breakthroughs have been presented for ASSBs with pouch cell format either in terms of energy density or cycle stability. Therefore, both academia and industry are trying to scale up the technology from mold cell research to achieve practical electrochemical performance of ASSBs with pouch cell format.

The cathode material accounts for over 40% of the total cost of lithium battery and influences the overall energy density during the electrochemical processes.^[^ ^11^ ^]^ In the past few years, two classic type of cathode, intercalation compounds and conversion materials have developed a lot, strategies on improving their rate capability, stability, and cycle life, are the focus of the research currently.^[^ ^12^ ^]^ The electrochemical mechanism of the former ones is that the lithium ions are embedded in the material architecture for ion storage, while the latter ones are conversion materials that generate other phases by reacting with lithium ions to release the capacity.^[^ ^13^ ^]^ Nevertheless, the battery cycle performance is not enough to support the increasing demands based on LEs.^[^ ^14^ ^]^ The main reason is that the organic solvent to corrode the electrode material, thus generating solid electrolyte interphase (SEI) with organic and inorganic phases of cathode electrolyte interphase (CEI).^[^ ^15^ ^]^ However, these issues can be remitted by ASSBs because of the solid‐solid reaction pathways. For instance, Nazar et al. achieve more than 3000 cycles with 80% capacity retention at room temperature when using LiNi_0.85_Co_0._1Mn_0.05_O_2_ cathode, providing ASSBs with great hope to surpass the performance of existing LIBs.^[^ ^16^ ^]^ However, scaling up those strategies for the application of pouch cell system is difficult due to lots of other scientific and engineering problems.

Herein, in this review, a roadmap for analyzing the developed cathode materials in solid‐state batteries particularly for pouch cells is provided, aiming to show their advantages and disadvantages. First, we elaborate two types of cathode materials used in the preparation of ASSBs, including intercalation compounds and conversion materials. Then the universal problems such as mechanical problem and electrochemical instability, and the corresponding improving strategies of these materials in the preparation of pouch cells are dissected. In addition, the inorganic solid electrolytes including the sulfide solid electrolytes and halide solid electrolytes are briefly analyzed. At the end of this review, the development of inorganic solid‐state pouch cells is summarized and prospected, aiming to provide a guide for the practical design of ASSBs and accelerate their practical applications.

## 2 Cathode Design for Solid‐State Pouch Cells

Laboratory‐scale mold cells are easily adapted as a platform with ultra‐small test area (< 1 cm^2^) to perform the most basic scientific investigation.^[^ ^17^ ^]^ Based on recent electrochemical data, ASSBs can achieve thousands cycles easily, which is enough for commercial use as only considering the cyclic stability.^[^ ^18^ ^]^ However, once considering to scale up the related strategies, significant differences in solid‐state pouch cells including capacity, cycles, reliability, and external pressures are revealed, giving rise to a set of new challenges. In this section, we mainly focus on the challenges and the state‐of‐the‐art progresses in the cathode design, aiming to provide guidelines for future development of the solid‐state pouch cells. Finally, the research directions for high‐performance cathodes are suggested based on the recent advances.

### 2.1 The Classic Cathode Materials Applied for the Pouch Cells

**Figure 1:**
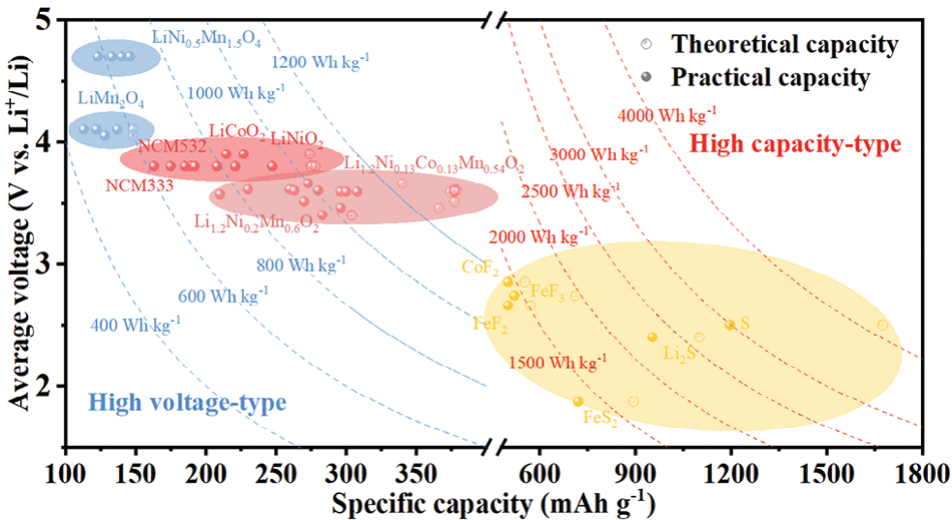
Properties of existing cathodes based on intercalation compounds and conversion materials.

As shown in **Figure** **1**, the existing cathode active materials (CAMs), which are very promising to be used in ASSBs, could be divided into two types. One is the intercalation compounds (>3.5 V) with intercalation mechanisms based on layer structure (e.g., LiM_x_O_2_ (M = Ni, Co, and Mn)), spinel structure (e.g., LiMn_2_O_4_) or olivine structure (e.g., LiFePO_4_), while the other one is the conversion materials with high specific capacity (> 500 mAh g^−1^) such as metal chalcogenides, metal disulfide, and sulfur, etc.^[^ ^1^, ^19^ ^]^ The former occupies the main hotspot in current research and is the most promising candidate to date, because the assembled solid pouch cells have the potential to achieve more than 300 Wh kg^−1^ cell‐level energy density. The latter still needs to overcome lots of basic issues such as how to increase the mass content of active materials in the cathode with more than 80% to compete with the energy density of intercalation compounds.

#### 2.1.1 Intercalation Cathode Materials

**Figure 2:**
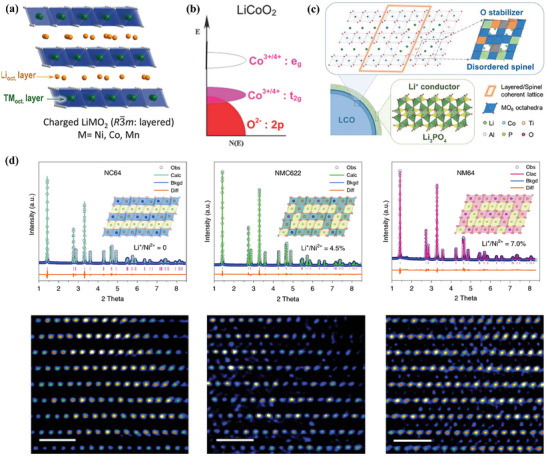
a) Schematic illustration of crystal structure of layered‐type cathode materials. Reproduced with permission.^[^ ^21^ ^]^ Copyright 2014, American Chemical Society. b) Energy diagrams of LiCoO_2_. Reproduced with permission.^[^ ^22^ ^]^ Copyright 2008, Royal Society of Chemistry. c) Schematic illustration of the LATP coating layer growth mechanism. Reproduced with permission.^[^ ^23b^ ^]^ Copyright 2020, Wiley‐VCH. d) Quantitative atomic occupancy analysis via HEXRD refinements and aberration‐corrected high‐resolution TEM. Reproduced with permission.^[^ ^28^ ^]^ Copyright 2021, Springer Nature.

Intercalation cathode materials usually have relatively low specific capacity but can achieve a high energy density of > 300 Wh kg^−1^ due to the high discharge voltage, which is highly attractive for practical applications.^[^ ^20^ ^]^ A typical material is LiCoO_2_, which is the earliest commercialized and most widely used LIBs CAMs. As shown in **Figure** **2**a, LiCoO_2_ is an α‐NaFeO_2_ type ordered layered structure with a hexagonal crystal system, belonging to the R‐3m space group.^[^ ^21^ ^]^ During the battery operation, the Li^+^ can be reversibly (de‐) intercalated into the inner of LiCoO_2_, giving rise to high rate performance and excellent structural stability. However, as shown in Figure 2b, the overlap in the position of Co‐t_2g_ and O‐2p band leads to the formation of (O_2_)^2−^ and release of O from the crystal lattice, causing the collapse of crystal structure if the charging voltage exceeds 4.6 V.^[^ ^22^ ^]^ Meanwhile, LiCoO_2_ undergoes a series of phase transitions when the Li^+^ is deintercalated during the charging process.^[^ ^23^ ^]^ Surface coating is a very common technique to improve its electrochemical stability. For instance, Li et al. improved its stability via surface coating with Li_1.5_Al_0.5_Ti_1.5_(PO_4_)_3_ (LATP) in order to generate a high‐voltage stable surface layer (Figure 2c).^[^ ^23b^ ^]^

Beside LiCoO_2_, LiNiO_2_ is also featuring an α‐NaFeO_2_ type layered structure.^[^ ^24^ ^]^ Its theoretically discharge specific capacity is 275 mAh g^−1^, and the reversible specific capacity can reach more than 180 mAh g^−1^.^[^ ^25^ ^]^ Nevertheless, the similar ionic radius of Ni^2+^ with Li^+^ cause the Ni^2+^ to occupy the Li^+^ sites during the charging and discharging process, resulting in the structural phase transition of LiNiO_2_ and the limitation of the cycling performance.^[^ ^26^ ^]^ Another similar material is LiMnO_2_ with slight difference, which presents a high theoretical capacity of 285 mAh g^−1^, but its cycling performance is poor due to the O atoms that are arranged with a torsional altered tetragonal dense stack in a layered rock salt structure.^[^ ^27^ ^]^ In addition, LiMnO_2_ is structurally unstable after Li^+^ deintercalation and will slowly shift to a spinel‐type LiMn_2_O_4_, at which Li^+^ will enter the Mn^3+^ layer, causing capacity decay.

The replacement of Co by Mn and Ni derivates the LiNi_1‐y‐z_Mn_y_Co_z_O_2_ (NMC) materials. The emergence of NMC materials effectively overcomes the disadvantages of LiCoO_2_, LiNiO_2_, and LiMnO_2_. In NMC materials, the valence states of Ni, Co, and Mn are +2, +3, and +4, respectively. The electrochemically active Ni^2+^ and Co^3+^ ensure the high discharge specific capacity of NMC materials with more than 180 mAh g^−1^ according to different atomic ratio of Ni, Co, and Mn.^[^ ^29^ ^]^ Moreover, the electrochemical inertness of Mn^4+^ acts as a skeleton to stabilize the crystal structure and enhances the stability of the materials. As present in Figure 2d, the presence of Co^3+^ significantly inhibits the mixing of Li^+^ and Ni^2+^ in NMC materials,^[^ ^28^ ^]^ allowing the NMC systems with plentiful advantages such as high volumetric energy density, wide operation voltage range, and low price. The NMC materials face two main challenges when paired with the SEs. One is that SEs are electrochemical oxidized by NMC when operating at high voltage, resulting in the formation of high resistance CEI. The other is the significant volume change during cycling, which causes aggravation of the contact problems and the mechanical degradation of secondary particles.

**Figure 3:**
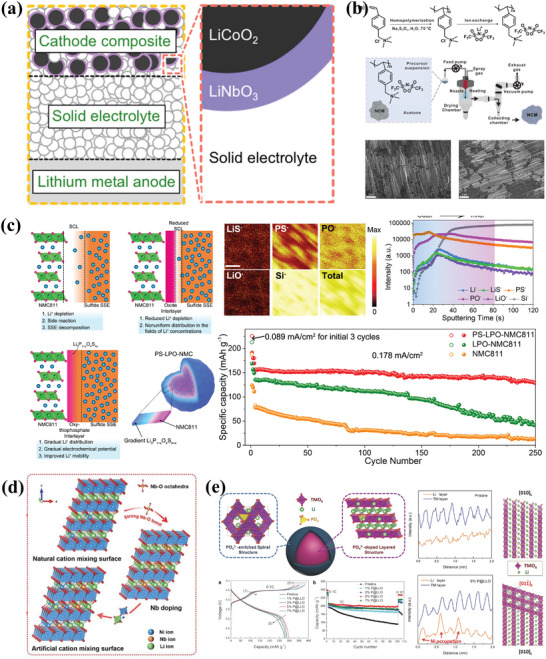
a) Schematic illustration of the CAMs/SEs contact in ASSBs. Reproduced with permission.^[^ ^35^ ^]^ Copyright 2020, Elsevier. b) Schematic illustration of the process for spray coating of NCM particles with PVBTA‐TFSI and FIB‐SEM images of composites cathode after cycle. Reproduced with permission.^[^ ^44^ ^]^ Copyright 2023, Wiley‐VCH. c) Diagram of NMC811 cathodes with gradient Li_3_P_1+x_O_4_S_4x_ coating and cycle performance of the PS‐LPO‐NMC811 cathode. SCL stands for space‐charge layer and PS‐LPO‐NMC stands for gradient Li_3_P_1+x_O_4_S_4x_‐coated NMC811. Reproduced with permission.^[^ ^45^ ^]^ Copyright 2023. Springer Nature. d) Schematic diagram of doping mechanism. Reproduced with permission.^[^ ^46^ ^]^ Copyright 2023. Elsevier. e) The structure diagram of cathode with PO_4_^−^ doped and corresponding cycle performance. Reproduced with permission.^[^ ^47^ ^]^ Copyright 2016, Wiley‐VCH.

As depicted in **Figure** **3**a, buffer layer is necessary for the stability of the CEI and LiNbO_3_ layer is one of the common choices. Deposition technologies such as magnetron sputtering,^[^ ^30^ ^]^ mechanical mixing,^[^ ^31^ ^]^ chemical vapor deposition (CVD),^[^ ^32^ ^]^ dry deposition,^[^ ^33^ ^]^ atomic layer deposition (ALD),^[^ ^34^ ^]^ wet‐chemical methods,^[^ ^35^ ^]^ pulse laser deposition (PLD),^[^ ^36^ ^]^ and in situ reaction on the surface of cathode or electrolyte are proposed to avoid the direct contact between CAMs and SEs.^[^ ^37^ ^]^ These technologies effectively ensure the consistency of the interface between the cathode and the SEs and increase the contact area. However, the quality and reliability for large‐scale preparation still need to be studied, especially for the preparation of solid‐state pouch cells. In addition, surface coating materials such as ceramic components (LiNbO_3_,^[^ ^38^ ^]^ Li_3_PO_4_,^[^ ^36^ ^]^ Li_2_O‐ZrO_2_,^[^ ^39^ ^]^ AlF_3_,^[^ ^40^ ^]^ and Li_4_Ti_5_O_12_)^[^ ^41^ ^]^ and polymer materials (poly‐acrylonitrile,^[^ ^37^ ^]^ polyimide,^[^ ^42^ ^]^ poly (3,4‐ethylenedioxy‐thiophene))^[^ ^43^ ^]^ and poly ((4‐vinyl benzyl) trimethylammonium bis(trifluoromethanesulfonylimide))^[^ ^44^ ^]^ have been proposed to improve interfacial stability and enhance the structure stability of NMC materials. For instance (Figure 3b), Richter et al. reported a thin homogeneous coating of poly((4‐vinyl benzyl) trimethylammonium bis(trifluoromethanesulfonylimide) (PVBTA‐TFSI) on the surface of LiNi_0.83_Mn_0.06_Co_0.11_O_2_ cathode using a spray‐drying process to inhibit the formation of oxygenated species and alleviate the cracking of CAMs particle.^[^ ^44^ ^]^ With the protection of the PVBTA‐TFSI coating layer, no particle cracking was observed at the LiNi_0.83_Mn_0.06_Co_0.11_O_2_ cathode after 200 cycles. Similarly, Sun et al. proposed a gradient coating structure of LiNi_0.8_Mn_0.1_Co_0.1_ (NMC811) particles with thiophosphate salts (Li_3_P_1+x_O_4_S_4x_) by ALD of Li_3_PO_4_ on the materials surface, followed by in situ vulcanization to form a gradient Li_3_P_1+x_O_4_S_4x_ coating.^[^ ^45^ ^]^ This method enables precise regulation of the thickness and morphology of the coating layer (Figure 3c), thus inhibiting the structural degradation of the materials during the cycling process. As the result, the ASSBs achieve stable cycling for over 250 times at 0.178 mA cm^−2^ and maintains a high capacity retention rate of 80%. Despite significant progresses in the fabrication of coating layer on cathode surface, some key issues such as the cracking of coating layer during long‐term cycling process and high production cost remain to be resolved.

As present in Figure 3d, element doping can modify the intrinsic properties of the material to maintain structural stability and reduce capacity degradation during cycling.^[^ ^46^ ^]^ According to the different charge properties, the doping ions can be mainly classified into cations and anions. The introduction of Na^+^, K^+^, Mg^2+^, Al^3+^, La^3+^, Zr^4+^, and Nb^5+^, etc. into the NMC materials can inhibit the phase transition.^[^ ^48^ ^]^ For instance, it has been confirmed that Al^3+^ would increase the binding strength of transition metal (TM) ions to the O atoms, thus hindering the cation mixing and improving the structure stability of the NMC materials. Doping of O sites with anions or polyanions is another effective strategy to enhance the strength of TM‐O bonds. Zhao et al. developed a gradient PO_4_^3−^ polyanion doping strategy by co‐precipitation to trigger surface structural transformations, resulting in the simultaneous formation of spinel‐like surface nanolayers and layered core materials doped with polyanions.^[^ ^47^ ^]^ As shown in Figure 3e, the doping strategy combines the advantages of doping and surface coating to stabilizes the O closed‐packed structure and improves its oxidative stability. Although doping is an effective method to improve the electrochemically active of cathode, it is necessary to optimize the homogeneity of doping that determines the homogeneity of the electrode material.

**Figure 4:**
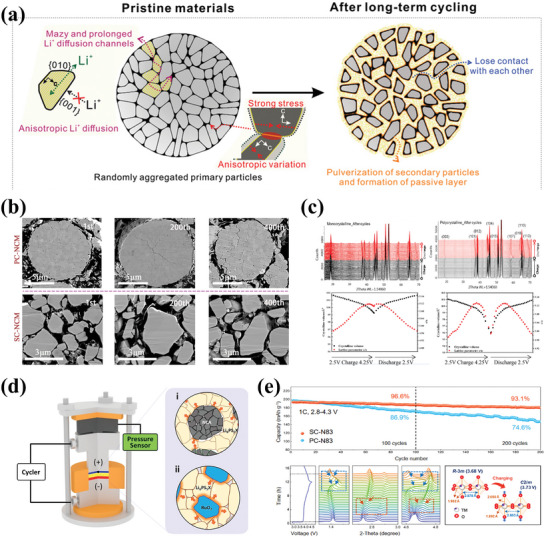
a) Schematic illustration of the structure and characteristics of NMC cathode. Reproduced with permission.^[^ ^49^ ^]^ Copyright 2019, Wiley‐VCH. b) Cross‐sectional SEM images of poly‐ (top) and single‐crystal (bottom) NMC cathode after different cycles. SC‐NCM stands for single‐crystal LiNi_0.83_Co_0.10_Mn_0.07_O_2_ and PC‐NCM stands for polycrystalline Ni‐rich cathode materials. Reproduced with permission.^[^ ^50^ ^]^ Copyright 2021, Elsevier. c) The in‐situ XRD patterns of single‐ and poly‐crystal NMC materials after 600 cycles. Reproduced with permission.^[^ ^51^ ^]^ Copyright 2021, Elsevier. d) Schematic of a pressure sensor. Reproduced with permission.^[^ ^54^ ^]^ Copyright 2021, Wiley‐VCH. e) The structure analysis of single‐crystal NMC cathode and the corresponding cycling performance. SC‐N83 stands for single‐crystal Ni‐rich LiNi_0.83_Co_0.12_Mn_0.05_O_2_, PC‐N83 stands for polycrystalline analogue. Reproduced with permission.^[^ ^53^ ^]^ Copyright 2021, American Chemical Society.

Apart from the phase transitions, the layered‐type CAMs undergo significant volume changes during (de‐)lithiation processes, which lead to the contact loss between CAMs and SEs and the rupture of CAM secondary particles, as depicted in **Figure** **4**a.^[^ ^49^ ^]^ Such a secondary particles are not a significant hazard in LEs‐based LIBs as liquid can easily penetrate the newly formed fissures. However, it is a disaster in ASSBs due to the inherent characteristics of SEs. As present in Figure 4b, the absence of anisotropic volume change in single crystal materials guarantees a pristine shape, significantly suppressing the generation of microcracks.^[^ ^50^ ^]^ This morphological robustness inhibits the successive formation of high resistance CEI and stabilizes the electrochemical properties. Zheng et al. performed structural analysis of single‐ and poly‐crystal NMC811 cathodes after cycling by in situ XRD.^[^ ^51^ ^]^ As a result of their study, the lattice volume change of single‐crystal NMC811 cathodes during cycling is significantly smaller than that of polycrystalline NMC811 cathodes, revealing the superior structural stability of single‐crystal NMC811 cathodes (Figure 4c). It should be noted that most of the studied single‐crystal NMC materials consisting of agglomerates of micro‐sized monolithic particles have not displayed a pure single‐crystal morphology because of the limitation of the synthesis method. Theoretically, such single‐crystal NMC CAMs are effective in preventing particles breakage, but the problem of contact loss with SEs remains due to the change in unit‐cell volume during cycling. For instance, Han et al. used high Ni single‐crystal CAMs paired with a halide solid electrolyte to achieve high capacity retention (96.8% after 200 cycles) and high initial Coulombic efficiency (89.6%) (Figure 4d).^[^ ^51^ ^]^ Doerrer et al. reported that the composite cathode consisting of single‐crystal LiNi_0.83_Mn_0.06_Co_0.11_O_2_ particles and Li_6_PS_5_Cl SE exhibited excellent discharge capacity of 210 mAh g^−1^ at room temperature.^[^ ^52^ ^]^ Liu et al. studied the excellent structural stability of single‐crystal NMC materials by in situ XRD and in situ pair distribution function (PDF) analysis.^[^ ^53^ ^]^ They found that the unique Li embedding kinetics in single‐crystal NMC materials resulted in intermediate monoclinic crystal distortions and irregular H_3_ stacking during cycling process, generating an additional strain cushion to minimize cracking, thereby achieving high cycling stability (more than 93% capacity retention after 200 cycles, Figure 4e). It is worth noting that particle size plays an important role in the properties of CAMs. Studies to increase the active surface area and accelerate Li^+^ diffusion by reducing particle size are demonstrated by researchers. However, only when the charging cut‐off voltage exceeds 4.35 V causing secondary particle fracture does the performance of single‐crystal CAMs outperforms the poly‐crystal. In the LEs‐based LIBs, the capacity lost in the first cycle is related to the formation of SEI on the anode surface.^[^ ^55^ ^]^ Capacity loss bring by reaction on anode surface in liquid system reminds us that compensation for the excess Li used for anode side reactions is also needed to take into account in the design of the cathode composite.^[^ ^56^ ^]^

**Figure 5:**
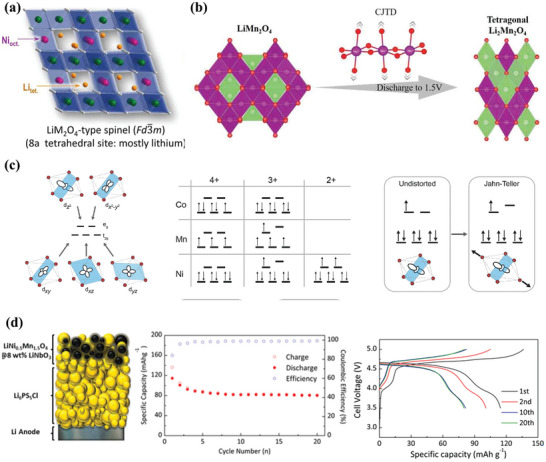
a) Schematic illustration of crystal structure of spinal‐type cathode. Reproduced with permission.^[^ ^21^ ^]^ Copyright 2014, American Chemical Society. b) Schematic illustration of Jahn‐Teller distortion in LiMn_2_O_4_ cathode. CJTD stands for cooperative Jahn–Teller distortion. Reproduced with permission.^[^ ^57^ ^]^ Copyright 2020, Wiley‐VCH. c) Crystal field spitting of d orbitals in an octahedral environment and Jahn‐Teller distortion due to specific electron configurations in LiMn_2_O_4_ cathode. Reproduced with permission.^[^ ^58^ ^]^ Copyright 2017, Wiley‐VCH. d) Diagram of Li‐LiNi_0.5_Mn_1.5_O_4_@8wt%LiNbO_3_ cell and corresponding cycling performance. Reproduced with permission.^[^ ^59^ ^]^ Copyright 2020, American Chemical Society.

Spinel‐type LiMn_2_O_4_ was first proposed by M. Thackeray and J. Goodenough et al. in 1983. As shown in **Figure** **5**a, LiMn_2_O_4_ belongs to a symmetric cubic crystal system, the F‐d3m space group. Its unit lattice has 32 O atoms, which maintain face‐centered cubic compact stacking, with the Li^+^ occupying the tetrahedral site (8a) formed by O^2−^ stacking and the Mn^3+^/Mn^4+^ occupying the octahedral site (16a).^[^ ^21^ ^]^ In LiMn_2_O_4_, the empty O tetrahedral and octahedral are connected in co‐planar and co‐lateral manner. These empty spaces form 3D Li^+^ diffusion channels to ensure the excellent Li^+^ conductivity of materials. However, the disproportionation reaction of Mn^3+^ and the Jahn‐Teller effect lead to rapid capacity decay and low specific capacity of LiMn_2_O_4_ cathode (Figure 5b), making it difficult to meet the growing demand for energy storage.^[^ ^57^ ^]^ In the LiMn_2_O_4_ structure described above, the TM atom occupies interstitial positions coordinated by 6 O atoms forming a slightly distorted octahedron. As depicted in Figure 5c, the crystal field generated by the coordination of the octahedral O anions splits the d orbitals of the TM into the e_g_ and t_2g_ energy levels. During the lithiation process, the Mn ions with +4 valence accept the electrons provided by the Li to be reduced to the oxidation state with +3 valence.^[^ ^58^ ^]^ Since Mn^3+^ has only one electron in the doubly‐simplified e_g_ orbital, which results in an asymmetric distribution of electrons. At the same time, the electrons in the d_x2‐y2_ and d_z2_ orbitals exhibit different degrees of shielding to the Mn nucleus in different directions, while in order to stabilize the Mn^3+^ within the molecule, the Mn‐O bond is extended along one of the octahedral axes, and the perpendicularly oriented Mn‐O bond is shortened, which results in the elongation of the linear MnO_2_ arrangement along the axial direction and produces the Jahn‐Teller distortion. The Jahn‐Teller distortion will bring dramatic structural changes to the material and accelerate the destroying of the material structure. The researchers formed LiNi_0.5_Mn_1.5_O_2_ by partially replacing the Mn in LiMn_2_O_4_ with Ni to avoid the Jahn‐Teller distortion and disproportionation of Mn^3+^. The valence of Ni and Mn are +2 and +4 respectively. During the charging process, the Ni changes its valence from Ni^2+^ to Ni^4+^ while Mn^4+^ remains unchanged, resulting in a more stable structure. Moreover, the LiNi_0.5_Mn_1.5_O_2_ features a redox potential of up to 4.7 V (V versus Li^+^/Li), which ensures the high energy density of LEs‐based LIBs.

However, perpetuating the above advantages into ASSBs seems challenging based on the data reported in the available literature. In the case of sulfide‐based SE, for example, LiNi_0.5_Mn_1.5_O_2_ exhibits poor compatibility, leading to severe interface problems including degradation of SE, chemo‐mechanical failure, and formation of high resistance CEI etc.^[^ ^60^ ^]^ In order to solve the interface problem between LiNi_0.5_Mn_1.5_O_2_ cathode and SE, the conventional protective surface coating for LiNi_0.5_Mn_1.5_O_2_ CAMs has been adopted. Unfortunately, the performance of the LiNi_0.5_Mn_1.5_O_2_ cathode has not been significantly improved. For instance, Liu et al. employed a LiNbO_3_ for surface coating LiNi_0.5_Mn_1.5_O_2_ particles by wet chemistry and obtained a high initial discharge capacity of 115 mAh g^−1^ when combined with a high ionic conductivity Li_6_PS_5_Cl SE (the theoretical specific capacity of LiNi_0.5_Mn_1.5_O_2_ is 147 mAh g^−1^) (Figure 5d).^[^ ^59^ ^]^ However, the reversible discharge capacity drops to 80 mAh g^−1^ after only 20 cycles of battery operation. The high redox potential of spinel‐structured LiNi_0.5_Mn_1.5_O_2_ is a double‐edged sword, ensuring a high energy density of the battery while placing high demands on the electrolyte. The recent development of oxidation‐stable halide SEs may offer a viable solution for the realization of high‐voltage LiNi_0.5_Mn_1.5_O_2_ CAMs in ASSBs. Furthermore, the capacity decay mechanism and Jahn‐Teller distortion of LiNi_0.5_Mn_1.5_O_2_ cathode in ASSBs require further research. Meanwhile, although the application of SE reduces the dissolution of CAMs, the interfacial reaction between LiNi_0.5_Mn_1.5_O_2_ and SE should receive more attention.

#### 2.1.2 Conversion Materials

**Figure 6:**
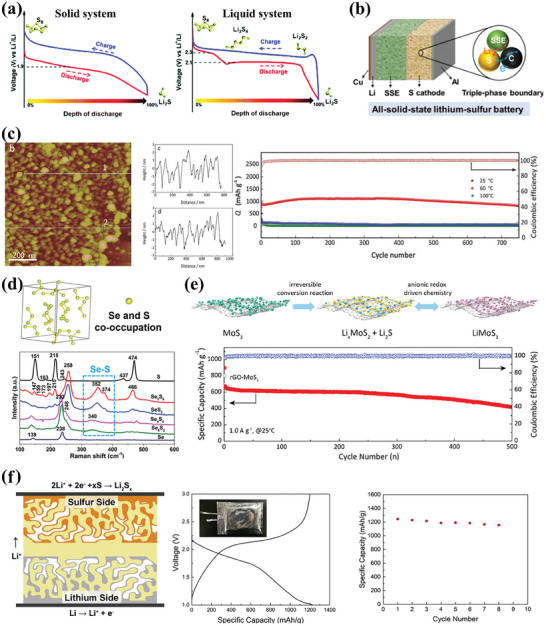
a) Typical charge/discharge voltage profiles of solid and liquid system Li‐S reactions. Reproduced with permission.^[^ ^64^ ^]^ Copyright 2020, Royal Society of Chemistry. b) Schematic illustration of SSLSBs. Reproduced with permission.^[^ ^64^, ^67^ ^]^ Copyright 2023. Springer Nature. c) The AFM image of rGO@S and corresponding cycling performance. Reproduced with permission.^[^ ^68^ ^]^ Copyright 2017, Wiley‐VCH. d) The structure and Raman spectra of Se_x_S_2_. Reproduced with permission.^[^ ^69^ ^]^ Copyright 2019, Wiley‐VCH. e) The reaction mechanisms of rGO‐MoS_3_ nanocomposites and corresponding cycling performance. Reproduced with permission.^[^ ^70^ ^]^ Copyright 2019, Elsevier. f) Diagram of Li‐S cell and corresponding cycling performance. Reproduced with permission.^[^ ^71^ ^]^ Copyright 2019, Elsevier.

The theoretical specific capacity of intercalation‐type CAMs is limited by the lattice position and the large voltage steps between different redox couple.^[^ ^61^ ^]^ By comparison, conversion‐type materials undergo significant phase transitions during cycling.^[^ ^62^ ^]^ The pivotal advantage of conversion‐type materials for CAMs is their high theoretical specific capacity. Typically, the elemental sulfur (S) based on conversion‐reaction delivers a high theoretical specific capacity of 1675 mAh g^−1^, and its reaction mechanism is different from (de‐) intercalation mechanism of conventional LIB.^[^ ^63^ ^]^ As presented in **Figure** **6**a, when the battery is discharging, the Li^+^ migrates from anode to cathode, and the S‐S bond of CAMs breaks to produce Li_2_S. The Li^+^ released from decomposition of Li_2_S returns to the anode and is deposited as metallic Li or embedded in the anode material during charging. This solid‐solid process is different with LEs, in which the interconversion of S to Li_2_S is a complex process involving a multi‐electron reaction.^[^ ^64^ ^]^ Among them, the dissolution of lithium polysulfides (LiPSs) in the electrolyte triggers the infamous “shuttle effect”, resulting in loss of active material and low Coulombic efficiency.^[^ ^65^ ^]^ In contrast, when the S cathode is matched to a ceramic‐based SEs, an alternative solid‐solid reaction path involving direct conversion between S and Li_2_S without the intermediate LiPSs is formed.^[^ ^66^ ^]^ Therefore, the differences in the reaction mechanisms of solid‐state lithium sulfur batteries (SSLSBs) based on different SEs put forward different requirements for the design of cathodes.

The use of conversion‐type cathodes is often limited by their inherent poor electronic or ionic conductivity. In most cases, composite cathode designs are widely accepted using a porous conductive framework as host, confining the active material to facilitate electrode reaction kinetics.^[^ ^17^, ^72^ ^]^ The conductive framework breaks the limits of charge transport kinetics within the CAMs particles and significantly facilitates the electrode reaction kinetics. The cathode design in ceramic SE‐based battery systems is more complex due to the slow kinetics of the one‐step solid‐phase reaction mechanism between S and Li_2_S during charge and discharge. Although the conductive carbon matrix as an S host significantly improves the charge transfer kinetics,^[^ ^73^ ^]^ the electrochemical reaction is still limited by the slow ion transportation.^[^ ^74^ ^]^ Therefore, the cathode design in ceramic‐based SSLSBs typically adopts a mechanical mixture of CAMs, SEs and conductive carbon (Figure 6b).

In general, smaller CAMs particles feature a higher active surface area and shorter inter‐particle diffusion paths, which facilitate the transport of electrons and ions in the cathode.^[^ ^75^ ^]^ Therefore, the development of nanoscale CAMs has been widely accepted as an effective strategy to improve the electrochemical performance of SSLSBs. For example, Xu et al. chemically deposited ≈2 nm S conformally coated on reduced graphene oxide (rGO@S), which greatly improved the electron and ion transport kinetics in the S electrode and reduced the interfacial resistance.^[^ ^68^ ^]^ As a result (Figure 6c), rGO@S cathode enables SSLSBs to provide a high reversible discharge specific capacity of 1340 mAh g^−1^ after 30 cycles and offers a significantly enhanced cycle stability of over 750 cycles at 1C. Interestingly, they also found that the rGO@S nanocomposite cathodes possess a uniform volume change during battery operation, which significantly reduced stress/strain and extended the cycle life of the battery. Zhang et al. prepared nano‐size S as the active material using a solvent exchange method and used carbon nanotubes (CNTs) as the conducting network of the composite cathode (S‐CNTs).^[^ ^76^ ^]^ The SSLSBs assembled with S‐CNTs composite cathode exhibits a high discharge capacity of 1140.9 mAh g^−1^ at 0.1C and maintains 100% capacity without decay over 400 cycles. Furthermore, the ASSLSBs still delivers a high discharge capacity of 834.3 mAh g^−1^ at 0.25 C even after 1000 cycles. In addition to the development of nanoscale active materials, a number of S‐containing compounds, which combine S with other metals or non‐metals, have been widely reported. For example, Li et al. prepared SeS_x_ solid solutions by introducing Se into S cathodes to improve electronic and ionic conductivity (Figure 6d).^[^ ^69^ ^]^ Moreover, the SeS_x_ cathode exhibits stability within 100 cycles at a current density of 0.4 A g^−1^. Zhang et al. also demonstrated the usage of MoS_3_ nanoparticles with excellent electrochemical properties as alternative S cathodes (Figure 6e).^[^ ^70^ ^]^ However, the disadvantages of these S‐containing compounds as cathode materials are also obvious, such as lower theoretical capacities and discharge voltages, which reduce the energy density of SSLSBs. In the study of Wachsman et al., they assembled solid‐state pouch cell with S as the cathode and Li metal as the anode, achieving a high reversible discharge specific capacity of 1244 mAh g^−1^ and a cell‐level energy density of 195 Wh kg^−1^(Figure 6f).^[^ ^71^ ^]^ However, several challenges remain including low percentage (< 60wt%) and loading (< 8 mAh cm^−2^) of S and high negative/positive (N/P) capacity ratio (> 5). How to overcome these issues will be the key to achieve high cell‐level energy density (> 500 Wh kg^−1^). It is worth noting that the interface problem between the S cathode and the SEs is exacerbated during the cycling. The large volume change of S and Li_2_S can cause the CAMs to separate from the ionic and electronic conductors, resulting in a large interfacial resistance and rapid capacity decay. Good contact between particles within the cathode layer by applying external pressure has been widely accepted as an effective strategy for solving interfacial problems. In the study by Suzuki et al., they used a composite cathode composed of S, SEs, and carbon replica.^[^ ^17a^ ^]^ The SSLSBs exhibits a high initial discharge specific capacity of over 2000 mAh g^−1^ under an applied pressure of 213 Mpa and still provides a discharge specific capacity close to 1500 mAh g^−1^ after 50 cycles, with a Coulombic efficiency close to 100% and a stable voltage polarization.

The conversion‐type S cathode cannot supply Li^+^ at the initial stage, resulting in the usage of metallic Li or lithiated compounds as the anode.^[^ ^77^ ^]^ The use of metallic Li significantly increases the energy density of lithium sulfur batteries (LSBs). Compared to commercial LIBs, LSBs deliver a theoretical energy density of up to 2600 Wh kg^−1^.^[^ ^78^ ^]^ However, this provides a major challenge for commercialization. Unstable Li deposition on the surface of Li metal and the formation of Li dendrites are critical issues, and the current research is only directed at inhibiting or retarding the growth of Li dendrites. The growth of Li dendrites during cycling can easily penetrate the SEs and lead to short‐circuiting of the battery. Although the high mechanical strength of ceramic‐based SEs can inhibit Li dendrite growth to some extent, those Li dendrites growing along grain boundaries can also lead to short circuits. Therefore, more attention needs to be paid to some lithiated conversion‐type CAMs, which may be the key to the commercialization of SSLSBs.

To achieve high‐performance solid‐state pouch cell, intercalation materials are more suitable than conversion materials for these main reasons: (1) In terms of battery performance: the capacity retention and rate performance of intercalation materials show obvious advantages over conversion materials; (2) From the perspective of commercialization: the production technology of intercalation materials is more advanced with faster commercialization process. However, the intercalation materials are still facing the following problems that might restrict their further development: (1) Safety issues: despite the protection of buffer layer, due to the high‐voltage condition, side reactions between intercalation materials and electrolyte are still unavoidable, resulting in heat and toxic gas generation; (2) Low energy density: the theoretical energy density of the intercalation material is significantly lower than that of the conversion material, and may not meet the application requirements in the future.

Compared with intercalation materials, conversion materials have two significant advantages: (1) Higher theoretical energy density, which is expected to break the energy density limit of existing battery systems; (2) Better electrochemical stability between the conversion material and the electrolyte, without obvious side reaction. Conversion materials may become a competitive choice for the cathode materials of solid‐sate pouch cell after solving two critical points: (1) Increasing the mass percent of CAMs: due to the low ionic and electronic conductivity of the conversion material, large amount of solid electrolyte and conductive additive is added, the content of active material is relatively low. (2) Accelerating the conversion reaction kinetics: compared with the liquid battery system, due to the higher interfacial resistance of the ASSBs and the sluggish kinetics of the solid‐solid conversion reaction, the reaction in the solid‐state system is insufficient, leading to inadequate utilization of CAMs and affects the rate performance of the pouch cell.

Therefore, intercalation materials are better choice for solid‐state pouch cell at this stage, while conversion materials are promising and may bring a new breakthrough for solid‐state pouch cell in the future.

### 2.2 Interfacial Contact Issues in Pouch Cell

The cathode in ASSBs usually consists of CAMs and SEs together with electronic conductive additives. However, there are a large number of key scientific problems that need to be solved inside the cathode structures, especially in the pouch cell system in which some key issues will be amplified. For instance, the electrode density can be well solved by cold pressing more than 300 MPa in the mold cells. However, it is difficult to ensure the complete density of the electrode in the pouch cells because of the weave of PTFE fibrosis, resulting in ultra‐high pressure that is required for electrode compaction. For instance, Samsung used a warm isobaric pressure (WIP) machine combined with 490 MPa pressure to allow the transfer of ions.^[^ ^10a^ ^]^ Therefore, in this section, a series of common challenges at the materials level in mold and pouch cells are first discussed, which needs to ongoing investigate the mechanical contact loss, chemical and electrochemical instability between the solid‐state cathode and the SEs.

#### 2.2.1 Mechanical Contact Loss

**Figure 7:**
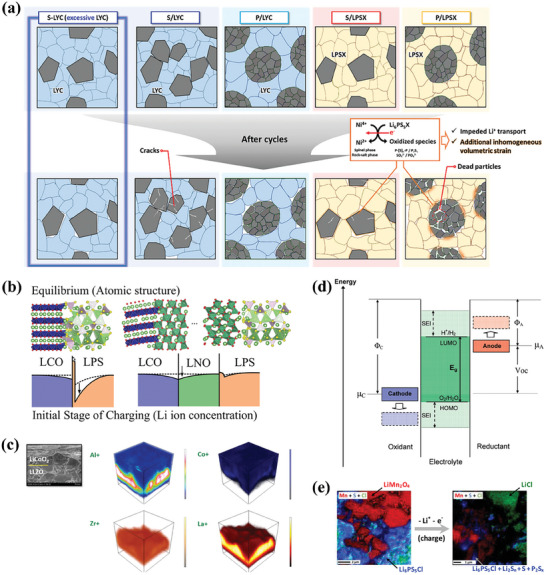
a) Schematic illustrating the different microstructural and interfacial evolutions in the LiNi_0.88_Co_0.11_Al_0.01_O_2_ (NCA) cathode in ASSBs. LYC stands for Li_3_YCl_6_, LPSX stands for Li_6_PS_5_Cl_0.5_Br_0.5_. Reproduced with permission.^[^ ^54^ ^]^ Copyright 2021, Wiley‐VCH. b) Surface structures of the LiCoO_2_(110)/Li_3_PS_4_(010) and schematic illustrations of the interfacial Li concentration. LCO stands for LiCoO_2_, LPS stands for Li_3_PS_4_, LNO stands for LiNbO_3_. Reproduced with permission.^[^ ^79^ ^]^ Copyright 2014, American Chemical Society c) The 3D elemental maps of the composite LiCoO_2_/Li_7_La_3_Zr_2_O_12_ interface determined by TOF‐SIMS. Reproduced with permission.^[^ ^80^ ^]^ Copyright 2016, American Chemical Society. d) Schematic of the relative electron energies. Reproduced with permission.^[^ ^81^ ^]^ Copyright 2010, American Chemical Society. e) Schematic illustration of the interface stability in ASSBs. Reproduced with permission.^[^ ^82^ ^]^ Copyright 2017, American Chemical Society.

Adequate contact between electrolyte and electrode in a battery system is an effective guarantee of rapid ion transport. However, in ASSBs systems, the mechanical problems between the SEs and CAMs involve many factors. Different from the flowable LEs that enable well wetting of the interface on active materials, affording fast and efficient Li^+^ migration to provide high capacity, the point‐to‐point contact in solid system undoubtedly limits the diffusion of Li^+^ at the electrode/electrolyte interface, as shown in **Figure** **7**a. Therefore, the interface impedance in ASSBs is much larger than that in liquid batteries and the capacity cannot be sufficiently released. In addition, most CAMs may undergo phase transitions accompanied by large volume changes during repeated charging and discharging processes, which will lead to further mechanical contact loss between the SEs and the CAMs. Besides, the stress accumulation of active materials leads to the cracking of secondary particles during the batterie's operation, causing in the battery failure.

#### 2.2.2 Electrochemically Contact Instability

In all‐solid‐state pouch cell system, the release of capacity becomes more difficult than mold cells because there are more contact interfaces of cathode and electrolyte inside the battery, resulting in severe chemical and electrochemical instability. As shown in Figure 7b,c, the space‐charge layer (SCL) and element diffusion are typical cases for the complex interfacial layer with the chemical instability. As shown in Figure 7b, electrons can easily migrate from the electrolyte to the cathode due to the large chemical potential difference between them, giving rise to the Li^+^ near the interface diffuse toward the direction of electric field. After migration of Li^+^ under the electric field, changing of Li^+^ concentration forms a Li^+^ depletion zone, results in a large interfacial impedance between the CAMs and the SEs.^[^ ^79^ ^]^ Although researchers have demonstrated that the SCL was the main cause of excessive impedance for solid‐state pouch cells, the mechanism about the chemical formation process of the SCL is still poorly understood.

Figure 7c shows the element diffusion that usually occurs at the interface between the oxide‐based SEs and the oxide‐based CAMs. In general, high temperature co‐sintering is adopted to densify the composite cathode for a good interfacial contact. In this process, high temperature can induce mutual diffusion of element. The interfacial chemistry properties of LiCoO_2_/Li_7_La_3_Zr_2_O_12_ have been reported by Goodenough et al.^[^ ^80^ ^]^ They found the obvious Al diffusion from Li_7_La_3_Zr_2_O_12_ to LiCoO_2_ during the co‐sintering process, even up to the opposite end of the LiCoO_2_ layer. In addition, the cubic phase Li_7_La_3_Zr_2_O_12_ at the interface partially transforms into tetragonal phase Li_7_La_3_Zr_2_O_12_ with poor ionic conductivity, thus causing a high interfacial resistance. Therefore, although high temperature helps to improve the interfacial contact, it may still degrade the electrochemical performance of ASSBs.

Different from chemical stability, the electrochemical stability reflects the ability of interface to maintain its original physicochemical properties under the action of electric field force. According to the classical front molecular orbital theory, the electrochemical instability between the cathode and the SE is related to the highest occupied molecular orbital (HOMO) level of the electrolyte, as shown in Figure 7d.^[^ ^81^ ^]^ When the HOMO level of the electrolyte is higher than the chemical potential (*µ_C_*) of the cathode, electrons are transferred from the electrolyte to the cathode, leading to oxidation of the electrolyte. It is important to emphasize here that the *µ_C_* of the cathode decreases as the cathode voltage increases, which means that the electrolyte is more likely to be oxidized when it is matched to a high voltage cathode. The electrochemical instability between the cathode and the electrolyte leads to a constant depletion of the CAMs and the generation of high impedance by‐products, as shown in Figure 7e.^[^ ^82^ ^]^ The ideal CEI should be chemically inert and electrically insulating to isolate the cathode from further reactions with the SE. At the same time, the CEI should feature a high ionic conductivity to ensure the stable operation of the battery. However, the oxidation of SE usually results in the formation of phases with poor ionic conductivity. In the case of thiophosphate‐based SEs, for instance, the oxidation products of phosphite, sulfates, and polysulfide all exhibit poor ionic conductivity. This inadequate CEI leads to an increase in interfacial resistance thereby hindering charge transfer.

In summary, the ideal cathode in an ASSBs systems should possess a stable and continuous ion/electron transport network to ensure fast and efficient charging and discharging of the battery. In addition, it should contain sufficient reversible active Li^+^ to ensure stable battery operation. At the same time, chemical and electrochemical stability of CAMs are necessary for the safe operation of the battery over a high voltage and wide temperature range. Finally, the ideal CAMs should be environmentally friendly, abundant in raw materials, and low‐cost.

### 2.3 Fabrication of Sheet‐Type Cathode in Pouch Cells

**Figure 8:**
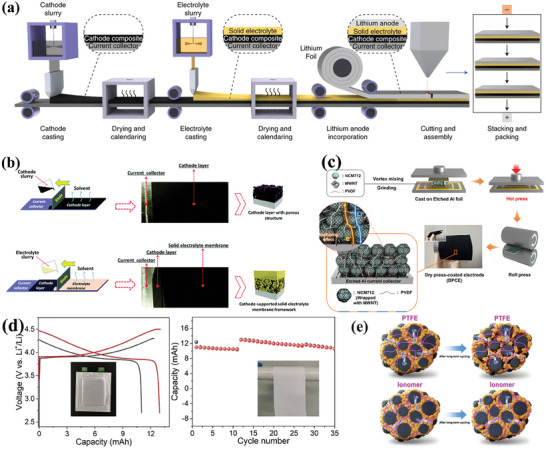
a) Schematic of large‐scale manufacturing of ASSBs. Reproduced with permission.^[^ ^86^ ^]^ Copyright 2020, Springer Nature. b) The sheet‐type electrode preparation by wet chemistry method. Reproduced with permission.^[^ ^83^ ^]^ Copyright 2020, Royal Society of Chemistry. c) The sheet‐type electrode preparation by dry electrode technology. Reproduced with permission.^[^ ^87^ ^]^ Copyright 2020, Springer Nature. d) The cycling stability of solid‐state LiCoO_2_ pouch cell based dry electrode technology. Reproduced with permission.^[^ ^88^ ^]^ Copyright 2021, Wiley‐VCH. e) Illustrations of the morphological changes experienced by composite cathodes induced by cycling. Reproduced with permission.^[^ ^89^ ^]^ Copyright 2022, American Chemical Society.

The prerequisites for pouch cells need the cathode architecture with sheet‐type structure to achieve Ah‐level high capacity. As shown in **Figure** **8**a, the roll‐to‐roll machinable sheet electrodes distinguish pouch cell from the mold cell. The sheet‐type composite cathodes are generally prepared by wet chemical methods (Figure 8b), in which polymer binders play an important role.^[^ ^83^ ^]^ For instance, Liang et al. investigated the effects of different types of binders (e.g., polyethylene oxide (PEO), polyvinylidene fluoride (PVDF), and carboxyl‐rich polymer (CRP)) on the electrochemical performance of LiCo_2_O_4_‐based ASSBs.^[^ ^84^ ^]^ The results show that the CRP binders with carboxyl groups exhibit a strong bond to the CAMs, ensuring a high stability of the cathode structure. In addition, the CRP can be used as a coating material to alleviate the side reactions between the cathodes and the SEs due to the superior oxidative stability of CRP. Based on these qualities, LCO‐based ASSBs using CRP binders can operate stably for more than 1000 cycles with a capacity retention of > 60% at the cut‐off voltages of 2.7–4.3 V. In general, binders are essential for the preparation of sheet electrodes in ceramic‐based ASSBs, but the available binders are severely limited. It is because that the common polar solvents used to dissolve the binders are highly reactivity with sulfide‐based and halide‐based SEs. In recent years, there has been an effort to screen solvents and binders that offer favorable compatibility with inorganic sulfide‐based SE. For instance, Zhang et al. demonstrated for the first time the compatibility of Li_10_GeP_2_S_12_ (LGPS) in n‐hexane with weak polarity by screening organic solvents with different polarities.^[^ ^85^ ^]^ They achieved the large‐scale preparation of sheet‐type S cathodes by a simple slurry coating process. The assembled solid‐state Li‐S pouch cells provides areal discharge capacity of presents 2.3 mAh cm^−2^ with high discharge specific capacity over 1150 mAh g^−1^ and excellent cycling stability.

In addition to the limitations of the binder and solvent, traditional wet chemistry process also faces challenge in the preparation of thick electrodes in ceramic‐based ASSBs without ion transport blockage. To overcome this challenge, solvent‐free dry electrode technology offers a “powder‐to‐film” pathway with the potential to simplify the production, receiving widespread attention from academia to industry (Figure 8c).^[^ ^87^ ^]^ Hippauf et al. prepared free‐standing NMC sheet electrodes with a high area loading of 6.5 mAh cm^−2^ using a solvent‐free dry film process.^[^ ^90^ ^]^ They fabricated the sheet electrodes by pre‐dry mixing of CAMs, conductive carbon, Li_6_PS_5_Cl and fibrous polytetrafluoroethylene (PTFE) binder followed by simple cold pressing. Notably, the all‐solid‐state pouch cells exhibit excellent cycling stability over 100 cycles without external pressure with a capacity retention rate of over 90%. It confirms the potential and functionality of the dry film process. Ludwig et al. employed a dry powder spraying process to prepare electrodes instead of the traditional slurry casting process, which offers better economic efficiency and greater bonding strength to the collector fluid.^[^ ^91^ ^]^ The electrodes prepared using the dry powder spraying process exhibit superior cycling stability over 50 cycles compared to the slurry casting process. In the recent work of Sun et al., they prepared LiCoO_2_ sheet‐type electrodes by dry film process and assembled an all‐solid‐state pouch cell with Li metal anode, which can provide a high capacity of 11 mAh at 0.03C and a high initial Coulombic efficiency of more than 90% (Figure 8d).^[^ ^88^ ^]^

The ion transport in thick electrodes is often limited by the polymeric binder in composite electrodes, as most polymeric binders possess a low ionic conductivity. To address this problem, Hong et al. designed an ionomer (poly (tetrafluoroethylene‐co‐perfluoro (3‐oxa‐4‐pentenesulfonic acid)) lithium salt) with high ionic conductivity as a binder for the preparation of thick electrodes in a solvent‐free dry film process (Figure 8f).^[^ ^89^ ^]^ The ionomer ensured fast Li^+^ transport and favorable interfacial contact between CAMs, conductive carbon and Li_6_PS_5_Cl SEs during cycling. As a result, the ASSBs with high area capacity (3.05 mAh cm^−2^) cathodes exhibits a high specific discharge capacity of 180.7 mAh g^−1^ at 0.1C and maintains 90% of their initial capacity after 300 cycles at 0.5C. Beside the intrinsic conductivity, the amount of binder should be in the right range as an excess of binder can hinder the transport of Li^+^ and electrons.

To date, the development of dry film processes offers a reliable scheme for the preparation of high‐performance thick electrodes for solvent‐sensitive SE. However, homogeneous mixing between the components of the electrode is difficult to achieve by the simple dry mixing method alone. In addition, the preparation methods for thick electrode should consider compatibility with the existing LIBs production facilities. In conclusion, the preparation of sheet composite cathodes will remain an important research topic for the future commercialization of SSLBs.

## 3 Brief Review of Electrolyte Chemistry for Solid‐State Pouch Cells

Inorganic solid electrolyte mainly includes oxides, sulfides, and halides based materials. Based on currently research progresses, only sulfide and halide electrolytes are expected to implement inorganic solid‐state pouch cells, because of the high Li ionic conductivity that can be obtained by cold pressing. Although oxide electrolytes represented by NASICONs, perovskites, and garnets have gone through a long process of development, they are still not applicable to solid‐state pouch cell for the following reasons: (1) Because of the poor contact between electrolyte and electrode, oxide‐based ASSBs can hardly cycle without adding liquid electrolyte or gel polymer electrolyte on the interface, which increases the risk of use and is contrary to the original intention of developing ASSBs, despite only in small amounts. (2) Oxide electrolytes have relatively low ionic conductivity. (3) High sintering temperature (≈1000 °C) for several hours is required in conventional process routes of NASICONs, perovskites, and garnets type electrolyte pellets, causing extremely high energy consumption when it comes to large‐scale production beyond pellets. (4) Mechanical fragileness of oxide electrolytes increases the difficulty of thin film (<20 µm) process, which limits the energy density and largely restricts the application of oxide electrolytes in solid‐state pouch cell. Though wet‐chemical deposited film and vacuum‐based techniques are expected to reduce the thickness, it is hard to make the membrane robust due to the poor mechanical properties of oxide electrolyte.^[^ ^92^ ^]^

Thus, in this section, we will mainly focus on the recent progresses with sulfide and halide‐based electrolytes, which are more reliable than oxide electrolyte for inorganic solid‐state pouch cells.

### 3.1 Sulfide Electrolyte

#### 3.1.1 Structure and Properties of Sulfide Electrolyte

**Figure 9:**
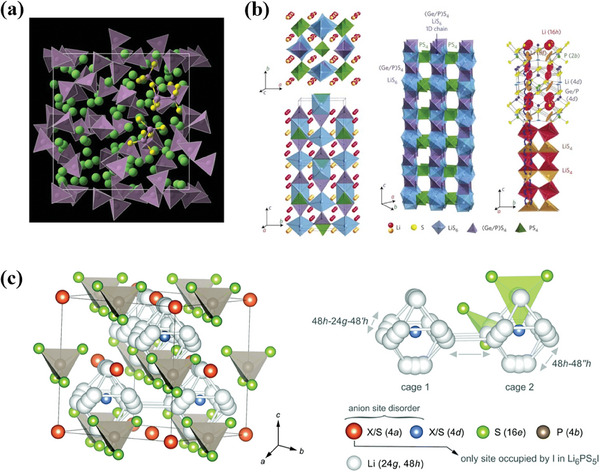
a) The framework structure of binary Li_2_S‐P_2_S_5_ glasses.^[^ ^96^ ^]^ Reproduced with permission. Copyright 2016, Springer Nature. b) The framework structure and lithium‐ion pathways of Li_10_GeP_2_S_12_. Reproduced with permission.^[^ ^94^ ^]^ Copyright 2011, Springer Nature. c) Crystal structure of argyrodite‐type Li_6_PS_5_X. Li ions are arranged octahedrally in the cubic lattice along with PS_4_^3^− tetrahedra, in the middle of the Li octahedra distribute some of the S^2−^ (4d) anions; the anions on 4a site are face centered cubic distributed, and lithium ions on two different positions (24 g, 48 h) form an octahedral arrangement. Reproduced with permission.^[^ ^97^ ^]^ Copyright 2019, Royal Society of Chemistry.

According to the precursor‐combinations, sulfide electrolyte can be divided into binary, ternary, and quaternary sulfide electrolyte. As shown in **Figure** **9**, binary Li_2_S‐P_2_S_5_ system is the earliest sulfide electrolyte explored by Mercier et al. and then developed to Li_2_S‐MS_2_ (M = Ge, Sn, Si) system by element doping to improve the ionic conductivity.^[^ ^93^ ^]^ The ternary SEs include Li_2_S‐P_2_S_5_‐MS_2_ (M = Ge, Si, Sn, Al, etc.) with a thio‐LISICON (lithium super ionic conductor) structure and a Li_2_S‐P_2_S_5_‐LiX (X = F, Cl, Br, and I) argyrodite structure. The LGPS exhibits high bulk ionic conductivity over 10 mS cm^−1^ owing to the 3D framework structure (Figure 9b).^[^ ^94^ ^]^ Different from LGPS, the lithium argyrodites (Li_2_S‐P_2_S_5_‐LiX) system has a cubic structure with S^2−^ and Cl^−^/Br^−^ at vertex and face center and possesses 3D lithium‐ion conduction pathways (Figure 9c).^[^ ^95^ ^]^ The PS_4_^3−^ tetrahedra distribute at mid‐point of each side, while two different lithium positions distribute on 24 g and 48 h together to form an octahedral. Together, the PS_4_^3−^ tetrahedra, face‐centered cubic at S^2−^, Cl^−^/B^−^, as well as lithium octahedron form a lithium silver structure.

**Figure 10:**
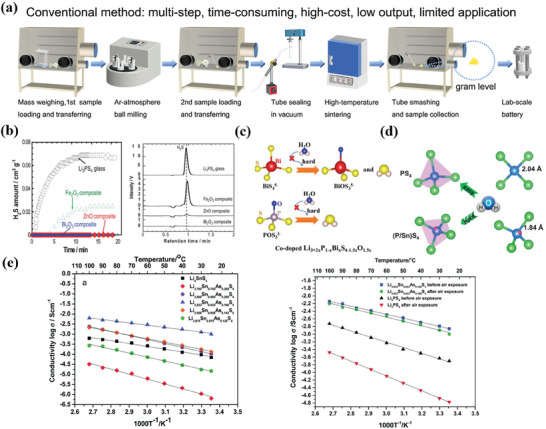
a) Preparation process of sulfide electrolyte in glove box, Ar‐atmosphere is required throughout the entire process which increase the time cost and process cumbersome. Reproduced with permission.^[^ ^101^ ^]^ Copyright 2021, Wiley‐VCH. b) The improvement on humid air stability of Fe_2_O_3_, ZnO, and Bi_2_O_3_ additives. Reproduced with permission.^[^ ^99^ ^]^ Copyright 2013, Royal Society of Chemistry. c) Chemical stability theory of Co‐doped Li_3.12_P_0.94_Bi_0.06_S_3.91_O_0.09_ and Optical pictures of the electrolyte tablet after immersed in water. Reproduced with permission.^[^ ^100^ ^]^ Copyright 2022, Wiley‐VCH. d) Schematic diagram of the difficulty of being oxidized by H_2_O for PS_4_ and (P/Sn)S_4_ tetrahedrons. Reproduced with permission.^[^ ^14b^ ^]^ Copyright 2020, Wiley‐VCH. e) The effect of doping amount of As on ionic conductivity. Reproduced with permission.^[^ ^102^ ^]^ Copyright 2014, Royal Society of Chemistry.

Sulfide electrolytes are considered to be one of the most promising electrolyte systems due to their excellent performance such as high ionic conductivity and good ductility.^[^ ^98^ ^]^ However, the drawbacks of sulfide electrolytes are further exposed once being employed to assemble pouch cells. As shown in **Figure** **10**a, the assembly processes need to be carried out in the glove box, which limits the feasibility of mass production and also increase the challenges for battery sealing. Many efforts have been done to improve stability of sulfide electrolyte against the humid air, including element doping, additives, structure design etc. For instance, Tatsumisago et al. studied oxide additives including Fe_2_O_3_, ZnO, and Bi_2_O_3_, which will not react with sulfide electrolyte.^[^ ^99^ ^]^ It is shown that adding 10 wt% of those oxides into binary glass electrolyte Li_3_PS_4_ will not affect the ion conductivity of composite electrolytes. After being exposed to air for 20 minutes, rarely H_2_S is generated from the composite electrolyte with ZnO and Bi_2_O_3_, suggesting that oxide additives significantly improve the air stability of sulfide electrolyte (Figure 10b,c).^[^ ^99^, ^100^ ^]^ As shown in Figure 10d, the P^5+^ existing in thiophosphate‐based electrolyte is a hard base with more prone to O^2−^. After replacing P^5+^ with As^5+^ and Sn^4+^, produced Li_4_SnS_4_ and Li_3_AsS_4_ show improved wet air stability.^[^ ^14b^ ^]^ However, the poor ionic conductivity is far below the requirement of ASSB. Accordingly, Liang et al. demonstrate that substituting As into Li_4_SnS_4_ improves the ionic conductivity, achieving the highest conductivity of 1.3 mS cm^−1^ in Li_3.833_Sn_0.833_As_0.166_S_4_ (Figure 10e).^[^ ^102^ ^]^

#### 3.1.2 Large Scale Fabrication of Electrolyte Membrane

**Figure 11. a):**
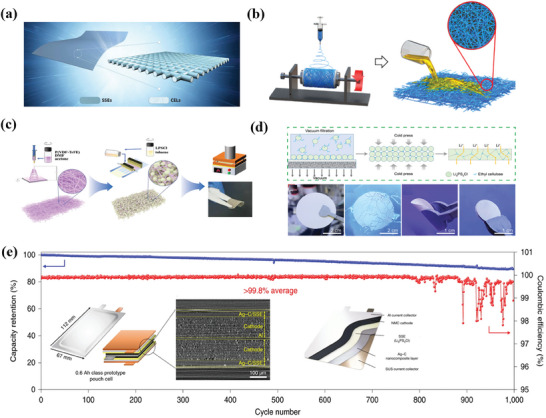
Schematic diagram of electrolyte membrane supported by polymer skeleton with good mechanical properties. CELs stands for cellulose. Reproduced with permission.^[^ ^104^ ^]^ Copyright 2021, Wiley‐VCH. b) Schematic diagram of the electrospinning used for fabricating electrolyte membrane skeleton and the slurry pouring process. Reproduced with permission.^[^ ^105^ ^]^ Copyright 2020. American Chemical Society. c) Schematic illustration for LPSCl@P(VDF‐TrFE) CSEs fabrication and the cycling performance of a pouch cell. Reproduced with permission.^[^ ^103^ ^]^ Copyright 2022, Wiley‐VCH. d) Schematic of the method for fabricating the thin SE membrane and the photos of the thin Li_6_PS_5_Cl SE membrane with and without ethyl cellulose. Reproduced with permission.^[^ ^106^ ^]^ Copyright 2021, Wiley‐VCH. e) Schematic diagram of the all‐solid‐state pouch cell using Ag‐C anode, X‐ray CT of the symmetric cell, and cycling performance of 0.6Ah Ag‐C/SSE/NMC prototype pouch cell. Reproduced with permission.^[^ ^10a^ ^]^ Copyright 2020, Springer Nature.

In addition to improving the air stability of sulfides, large scale fabrication without affecting the ionic conductivity of sulfide electrolyte is another challenge. Yao et al. develop a dry‐film process by mixing sulfide electrolyte (99.8 wt.%) and PTFE (0.2 wt.%) through ball‐milling and shearing. Hot calendaring was used for mechanical formation, thus obtaining a thin electrolyte membrane with 10 cm^2^ in area.^[^ ^15a^ ^]^ As shown in **Figure** **11**, it seems that solvent method based on polymer skeleton is more suitable for inorganic solid‐state electrolyte films. The uniformity of the solid electrolyte membrane has a great influence on its mechanical rigidity and ionic conductivity. The thickness of the electrolyte prepared by the solvent method is easy to control and the film formation is very uniform. The flexible electrolyte membrane prepared by the polymer skeleton has good folding performance and wide application scenarios (Figure 11a). Electrostatic spinning is another good choice (Figure 11b) because it can build a polymer framework involves large voids which is important for sulfide electrolyte to infiltrate. Nan et al. infiltrate LPSC‐toluene slurry into a P(VDF‐TrFE) electrospun membrane to obtain a composite electrolyte with good ductility and flexibility.^[^ ^103^ ^]^ The assembled pouch cell exhibits good cycling performance and high‐capacity retention (Figure 11c). PI non‐woven fabric was used as the scaffold for Lithium argyrodite electrolyte and the membrane is thermal stable up to 500 °C.^[^ ^105^ ^]^ Ethyl cellulose with amphipathic molecular structure and good binding capability can be used to produce a freestanding membrane (Figure 11d).^[^ ^106^ ^]^ Han et al. develop a solution‐process to fabricate membrane without polymer framework. They coated argyrodite powder slurry on PET film by doctor‐blade and then transferred it to NCM cathode by external pressure (50 Mpa).^[^ ^10a^ ^]^ In addition, they designed an Ag‐C composite anode that assist uniform and dendrite‐free lithium platting. The Ah‐class pouch cell exhibits the high energy density (>900 Wh l^−1^) and long cycle life (>1000 cycles), as shown in Figure 11e.

The stability of electrolyte membrane can also be improved by electrolyte structure design. To solve the problem of lithium dendrite penetration, Li et al. propose a multilayer design of sandwiched structure with a more‐stable solid electrolyte on the outside and a less‐stable electrolyte on the inside. According to their analysis, soon after the dendrite initiation and propagation, local mechanical constriction and strain field will suppress the Ge reduction. Such constrained decomposition operates as a self‐healing “concrete” that can heal the existing cracks. This effect is analogous to expansion screw effect and is verified in mold cell.^[^ ^107^ ^]^ Recently, Li et al. apply this multilayer design into solid‐sate pouch cell, the assembled Li‐NMC83 solid‐state pouch cell using a multilayer LPSC‐LPSP‐LPSC electrolyte membrane shows 80% capacity retention after 6000 cycles.^[^ ^108^ ^]^

Despite the above efforts, due to the instability of sulfide electrolytes, it is recognized that the main solvents are acetonitrile, chlorobenzene, and cyclohexanone for sulfide electrolytes.^[^ ^109^ ^]^ The introduction of those highly toxic chemical solvents in industrial mass production is not environmentally friendly. Solid‐polymer electrolyte consist of a homogeneous mixture of polymer and lithium salts, are flexible and soft with good ionic conductivity, they are alternative choice for pouch cell fabricating.^[^ ^6^ ^]^

### 3.2 Halide‐Based Electrolyte

**Figure 12:**
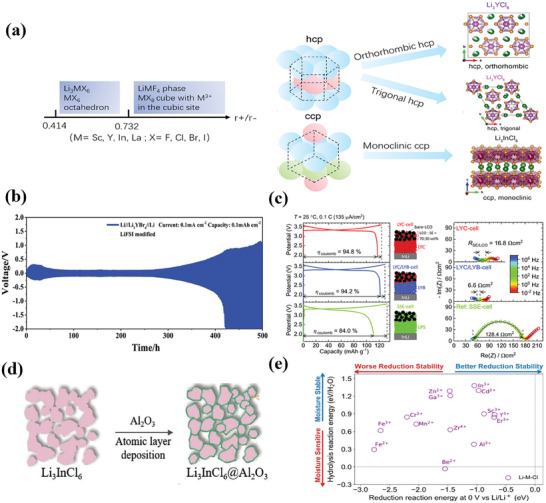
a) The main factors that determine the structure of halide‐based electrolytes. b) Li/Li_3_YBr_6_/Li cell that presents poor stability. Reproduced with permission.^[^ ^111^ ^]^ Copyright 2021, Wiley‐VCH. c) Better stability of halide‐based electrolyte showing better stability with cathode than sulfide electrolyte. Reproduced with permission.^[^ ^112^ ^]^ Copyright 2018, Wiley‐VCH. d) Coating Al_2_O_3_ layer by atom layered decomposition technique to improve the air stability of halide‐based electrolyte. Reproduced with permission.^[^ ^115^ ^]^ Copyright 2022, Wiley‐VCH. e) Effect of various elemental doping on moisture sensitivity and reduction stability. Reproduced with permission.^[^ ^113^ ^]^ Copyright 2020, Wiley‐VCH.

Halide‐based electrolytes have higher stability than sulfide electrolyte under high voltage. As shown in **Figure** **12**a, the ionic radius, polarity, and ion packing styles determine the structure of halide‐based electrolyte. For example, Li_3_MX_6_ has two kinds of anion sublattice structures. One is hexagonal close packing (hcp) and the other is cubic close packing (ccp). It is well‐known that halide‐based electrolytes have poor compatibility with lithium and even with lithium indium alloy anode (Figure 12b). Halide‐based electrolyte continuously reacts with lithium without the formation of a passivation layer.^[^ ^111^ ^]^ Therefore, bilayer electrolyte is designed, in which sulfide electrolyte contacts with anode while halide electrolyte contacts with cathode. As shown in Figure 12c, halide‐based electrolytes are expected to be stable toward high voltage transition metal oxide cathode materials including LiCoO_2_ and LiNi_0.8_Mn_0.1_Co_0.1_O_2_. Asano et al. use Li_3_YCl_6_ (LYC)and Li_3_YBr_6_ (LYB) as electrolyte to fabricate a battery with bare LiCoO_2_ cathode.^[^ ^112^ ^]^ The initial columbic efficiency of the LYC‐cell reaches 94.8% and that of the LYC/LYB‐cell reaches 94.2%, showing improvement compared with 84% in LiP_3_S_4_ cell. The interfacial resistance decreases from 128 Ω cm^−2^ for LiP_3_S_4_ cell to 6.6 Ω cm^−2^ for LYC/LYB‐cell and 16.8 Ω cm^−2^ for LYC‐cell.

For mass production in pouch cell application, most halide‐electrolytes are unstable in humid air. There are two main methods to increase the stablility of halides‐based electrolyte against humid air. One is to coat the electrolyte with a waterproof shell, and the other is to improve the intrinsic stability of halides by elemental doping or structure regulation. As shown in Figure 12d, coating electrolyte with waterproof layer such as Al_2_O_3_ cuts down its contact with humid air, which has been proved to be useful but not efficient for industrial production. Atom layered decomposition technique can also be used for coating Al_2_O_3_ on electrolyte, but it is difficult to be used for pouch cell application. In comparison, improving the intrinsic humid‐air stability through elemental doping or structure regulation seems to be a better choice (Figure 12e). Zhu et al. conclude a guidance for selecting moisture stable cations.^[^ ^113^ ^]^ In^3+^ is predicted to be both moisture air stable and electrochemical stable in halide‐based electrolyte. According to the calculation results, Sun et al. synthesized the Li_3_InCl_6_ electrolyte with high ionic conductivity of 1.49mS cm^−1^ and dry‐air stability. Although the performance degrades around 30% after being exposed to ambient air, the crystal structure can still recover after annealing. Sun et al. further studied the improved stability brought by In^3+^.^[^ ^114^ ^]^ According to their analysis, increase of In^3+^ ratio leads to the structural conversion from hexagonal‐closed‐packed (hcp) to cubic‐closed‐packed (ccp) by anion arrangement. The ccp structure and high In^3+^ ratio promote the formation of hydrated intermediates, enabling the heat triggered recovery.

Although halide‐based electrolytes are unstable in humid air, the electrolyte synthesis for Li_3_InCl_6_ and full cell fabrication can be carried under dry room conditions with dew point of −40°C, showing potential in large‐scale production. However, there is rarely report about halide‐based pouch cell at present. As mentioned before, the conductivity of halide‐based electrolytes is comparable to that of sulfides electrolytes. In future, finding suitable solvents and binders for wet chemistry synthesis and solving the problem of poor compatibility with lithium metal are necessary for pouch cell application. In addition, its price largely restricts its commercialization. The expensive anhydrous Li_2_S and high energy consumption during high‐energy ball milling or high temperature melting contribute to the high cost of argyrodite‐type electrolyte. Moreover, cations such as Sc^3+^ in halide‐based electrolyte mostly have low abundance, thus greatly increase the cost. Recently, Ma et al. synthesis Li_1.75_ZrCl_4.75_O_0.5_ oxychloride solid‐state electrolyte with cost‐effective precursor, which exhibits high ionic conductivity of 2.42 mS cm^−1^ with the cost of $11.60 kg^−1^.^[^ ^116^ ^]^ Until now, LIBs have developed for a long time with cost of $132 per kWh today, and will decrease to $50 by 2030. Overall, inorganic electrolyte is not a good option for cost‐effective and large‐scale manufacturing at present.

## 4 Conclusions and Outlook

In summary, comprehensive discussion about the cathode materials applied in the ASSBs especially for the pouch cell format, has been provided in this review, covering the reliable design, essential issues, and bipolar stacking technology. Initially, all the currently used materials or strategies are investigated in the mold cells and rarely discussed in the pouch cells with low discharge capacity (< 50 mAh). However, the ASSBs with mold or pouch cell formats are two completely different structure, and the related strategies from mold cells cannot be easily transferred to pouch cell format. For instance, the cyclic stability of Li‐S coin cell has completely exceeded 1000 cycles, but that of Ah‐level Li‐S pouch cells is still less than 100 cycles. Indeed, it is important to investigate the basic scientific questions based on mold cells, but more practical pouch cell formats should be included in the research program. Therefore, for the future development as well as evaluating the advanced materials for solid‐state pouch cell, the following factors are suggested to be considered:

*High‐Areal‐Capacity Thick and Robust Membranes*: The preparation of electrode films for pouch cells is categorized into two methods: the dry‐film techniques and the casting‐film techniques. The dry‐film technology involves PTFE fibrosis that is weaved inside the inner of materials, followed by roller pressing to prepare the electrode or electrolyte film. This method yields high ion conductivity in the prepared films and facilitates the high active material loading, which are the basic requirements to provide high energy density performance. However, this technology is still in its infancy with many limitations such as poor mechanical properties, difficulty in large‐scale production, and the lack of compatibility with existing lithium‐ion battery manufacturing processes. Compared to the dry method, the casting method offers compatibility with existing lithium‐ion battery manufacturing processes. However, it significantly affects the performance of electrode materials due to the high content of binders (> 5%) and the introduction of organic solvents during preparation. The high content of binder reduces the ionic conductivity of electrode films compared to only ≈0.5% PTFE binder in the dry electrodes. The introduction of polar solvents during the casting impacts the ionic conductivity electrolyte via forming a complex layer on the surface of solid‐state electrolyte and restricts the capacity release. Additionally, the casting technology requires gradual drying through heat treatment which results in numerous gaps during solvent volatilization. Therefore, developing dry electrode technology for high‐areal‐capacity thick and robust membranes used in the pouch cells may a better choice once the preparation processes are clarified.
*High Voltage Stable Interface*: The sulfide electrolytes tend to be easily oxidized to form a mix interphase with intercalation compounds such as LCO or NMC, leading to rapid performance degradation or failure of batteries. Thus far, numerous coating technologies on the surface of cathode or electrolyte materials have been developed. However, it remains unknown whether these technologies are suitable for large‐scale production with low energy consumption and compatible with pouch cell format. In contrast, halide electrolytes exhibit compatibility with intercalation cathode materials, giving rise to a new development path in the ASSBs. Nevertheless, further investigation is required to address the issues of poor ionic conductivity and high corrosion associated with halide electrolytes. Moreover, the reliability of those developed strategies is suggested to be tested in pouch cell system.
*Roll to Roll and Bipolar Stacking Technologies*: Currently, the reported technologies for all‐solid‐state pouch cells still involve manual stacking, in which the prepared electrode materials are rolled or coated onto the surface of current collector, cut to desired dimensions, and then matched with corresponding electrolyte films for stacking. However, the extremely pliable and mechanical fragile nature (typically only 50 µm thick) of those solid films give rise to significant challenges in terms of processing and transfer requirements in industrial settings. Therefore, it is crucial to develop mechanical strength of electrolyte films for rational stacking technologies. In addition, the challenges of bipolar stacking technologies are recommended to furtherly investigate.
*Acceptable Pressure*: The preparation of inorganic solid‐state pouch cells requires ultra‐high pressure to ensure a good contact between the electrode material and the electrolyte, thus reducing the internal porosity of the electrode and improving the ionic conductivities. Isostatic pressure is the only technology with more than 500 MPa to achieve the required pressure range so far. Isostatic pressure includes warm and hot isostatic pressure technology, but these technologies have not seen in the published articles, resulting in lots of knowledge blind spots about the preparation of solid‐state pouch cells. In addition, how to reduce the cell test pressure still needs to be explored, because high test pressure will cause lithium metal to pierce the SSEs. For this reason, reducing the pressure for both cell preparation and test is critical for the practical application of inorganic solid‐state pouch cells.

Finally, in recent years, China government has continually encouraged the development of solid‐state lithium batteries through policies. For example, the “New energy Automobile Industry Development Plan (2021‐2035)” issued by The State Council in October 2020 wrote that “accelerate the development and industrialization of all‐solid‐state power battery technology.” At the same time, the “energy‐saving and new energy vehicle Technology Roadmap 2.0” released by the China Society of Automotive Engineering also mentioned that “solid‐state battery research and development will be intensified”, and proposed “the layout of all‐solid‐state lithium‐ion and lithium‐sulfur batteries, and other new system battery R&D.” The overall goal of the battery performance is to reach 350 Wh kg^−1^ in 2025, 400 Wh kg^−1^ in 2030, and 500 Wh kg^−1^ in 2035. As early as 2019, Toyota Motor announced its cooperation with Panasonic to commit to the industrialization of ASSBs. Electric vehicles loaded with sulfide ASSBs have been used in the 2021 Tokyo Olympic Games. Panasonic, Hitachi, and other companies declared that the mass production of ASSBs would be completed by 2025. Nissan is also actively exploring sulfide‐based ASSBs technology, aiming to launch an electric vehicle with ASSBs in 2028. In addition, Solid Power in United States is also attracting more attention. The company has completed the installation of an automated production line for sulfide‐based ASSBs and started trial production. The production line will produce 300 solid‐state batteries per week, with an annual output of ≈15 000 cells. However, to date, the progresses of scientific exploration of basic principles related to ASSBs, the comprehensive performance of solid‐state pouch cells, the scale progresses of ASSBs and cost competitiveness are less than expected. In addition, the degree of information asymmetry in the ASSBs industry is more than expected. In order to validate the final roadmap and establish credibility, it is crucial to identify the key industrial players involved in ASSBs manufacturing to ensure that the prototype aligns with practical implementation and real‐world scenarios.

## Conflict of Interest

The authors declare no conflict of interest.
